# Visual function deficits in eyes with resolved endophthalmitis

**DOI:** 10.1038/s41598-021-81530-y

**Published:** 2021-01-27

**Authors:** Amithavikram R. Hathibelagal, Yasmeen Mulani, Vivek Pravin Dave

**Affiliations:** 1 Brien Holden Institute of Optometry and Vision Sciences, Kallam Anji Reddy Campus, LV Prasad Eye Institute, Hyderabad, India; 2 Smt. Kanuri Santhamma Center for Vitreoretinal Diseases, Kallam Anji Reddy Campus, LV Prasad Eye Institute, Hyderabad, India

**Keywords:** Preclinical research, Diagnostic markers, Signs and symptoms

## Abstract

To evaluate the changes in functional vision in patients with resolved endophthalmitis. This was a cross-sectional study. The study included 20 patients with resolved endophthalmitis and best-corrected visual acuity of 20/100 or better. Visual acuity (VA), contrast threshold (CT), red/green (RG) and yellow/blue (YB) colour vision and 15 Hz flicker modulation threshold (FMT) were assessed using standard psychophysical techniques. The median age was 54 years. The median visual acuity was 0.27 (~ 20/40—Snellen Equivalent) ((interquartile range [IQR]), 0.30) logMAR). The median log contrast threshold (CT) was − 1.13 (IQR, 0.36) log units (normative value for age-matched CT: − 1.61 log units). The median red/green (RG) and yellow/blue (YB) thresholds were 11.52 (IQR, 26.19) and 9.45 (IQR, 16.20) CAD units respectively, which were at least 5 times higher than age-matched normative RG and YB thresholds. The median central cone- mediated FMT was 17.64% (IQR, 23.40%), which was much higher compared to age-matched FMT (5.48% [IQR, 3.47]). Linear regression revealed significant relationship between contrast thresholds and foveal thickness (y = 0.001x−1.47, R^2^ = 0.20, p = 0.048). Though endophthalmitis may resolve with a good visual acuity, deficits in visual functions like chromatic discrimination, cone-mediated flicker and contrast sensitivity persist.

## Introduction

Endophthalmitis is characterized by marked inflammation of the intraocular tissues resulting from colonization by microorganisms that enter the eye directly (exogenous) or through the bloodstream from the site of infection from any other part of the body (endogenous) which can lead to severe vision loss, if not treated within 24 h^1,2^. Structural changes caused by endophthalmitis is typically captured by clinical examination and Optical Coherence Tomography (OCT), whereas the functional changes are captured only through only one visual function which is the best-corrected visual acuity and that remains the sole criterion for assessing the treatment outcomes. In patients with resolved endophthalmitis, although signs of macular oedema or epiretinal membrane persist, at least 50% of the eyes recover a visual acuity of 20/40 or better^3^. Despite the relatively good outcomes in terms of measured visual acuity, some of these patients often complain about the visual difficulties encountered in their daily activities and their quality of vision. This disparity between perception and clinical measure could be because visual acuity is measured in high-contrast and high-luminance test conditions. However, in a practical day-to-day routine, our natural environment spans over a continuum of different contrasts and luminances, the clinic-based conventional high-contrast visual acuity measurements alone may not capture all the functional deficits that the patients’ might experience^4^. Thus, there is a need to test other parameters of visual functions such as contrast sensitivity (spatial and temporal) and chromatic sensitivity, which are processed in the different domains of visual processing. Studying these visual functions would allow us a wider understanding of recovery of the various physiological mechanisms that mediate it and a finer comprehending of the profile of ocular complaints experienced by these patients, which can lead to better management. Each of these visual functions may act as additional useful outcome measures of treatment. Previously, it has been shown that visual functions such as spatial contrast sensitivity, chromatic sensitivity and cone-mediated flicker (temporal modulation) sensitivity are useful in the management and evaluation of treatment outcomes in retinal diseases^4^. In the current communication, we report the results of the various visual function parameters in eyes with resolved endophthalmitis.

## Materials and methods

### Subjects

**Figure 1:**
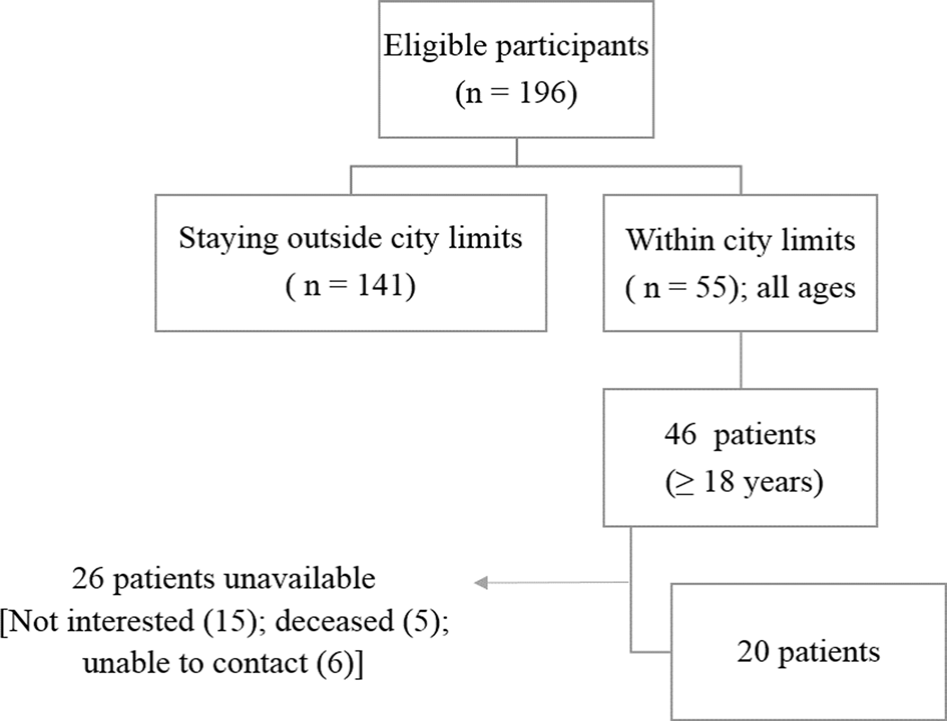
Flowchart showing recruitment process in the study.

This cross-sectional study was approved by the Institutional Review Board of Hyderabad Eye Research Foundation, L V Prasad Eye Institute, Hyderabad, India (Ethics Ref No. LEC-05-19-286). All research was performed in accordance with relevant guidelines/regulations, and an informed consent was obtained from all participants. A list of medical records of patients from LV Prasad Eye Institute, Hyderabad, who have recovered from endophthalmitis in the period 2015–2019 was obtained. The recruitment process for the patients with resolved endophthalmitis is shown in Fig. 1. The inclusion criteria for the study were defined as follows: 1. Age ≥ 18 years. 2. patients with resolved endophthalmitis (defined as the absence of any clinical signs of active inflammation as discerned by the treating physician). 3. Best-corrected VA of 20/100 (0.7 logMAR) or better in the affected eye, post-treatment. 4. No other ocular pathologies or complications, additional to endophthalmitis-related complications. 5. Any type of cataract, no greater than Grade II according to the Lens Opacification Classification System (LOCS)^5^. The treatment for all the patients included intraocular antibiotic (IOAB) injection along with Pars Plana vitrectomy in 80% (16/20) of the subjects. Written informed consent was obtained from all the participants before the study commencement. A comprehensive eye examination including visual acuity testing (using logMAR chart), refraction, slit-lamp biomicroscopy, applanation tonometry (Goldmann) and dilated fundus examination using indirect ophthalmoscopy was performed in all the participants to determine the eligibility before the recruitment of participants in the study. All the visual functions were measured monocularly only in the affected eye of all the cases.

### Visual acuity and contrast threshold measurement

Visual Acuity (VA) was assessed using COMPlog electronic system (COMPlog Clinical Vision Measurement Systems Ltd, London, UK) using the “thresholding” technique^6^. Contrast threshold function was measured using Pelli-Robson chart^7^ at a distance of 1 m with best—corrected vision under room illumination of 100 cd/m^2^.

### Flicker modulation and chromatic threshold measurement

Commercially available set-up Advanced Vision Optometric Test (AVOT) developed by City, University of London (City Occupational Ltd, U.K.) was used for assessing cone-mediated flicker threshold^8^ and red-green and yellow-blue chromatic threshold^4^. AVOT system employs a 10 bit high resolution display (24" IPS (in-plane switching)) LCD monitor (EIZO, Model ColorEdge CS2420; EIZO Corporation, Japan) and a laptop. The resolution of the display monitor is 60 Hz. A laptop was used by the examiner to present the stimulus on the display monitor. A black curtain separated the display monitor from the laptop. AVOT system includes a photometer (Mavo-Monitor USB, Gossen, Germany) for automatic calibration of the display monitor using a custom-built software.

Flicker-*Plus* module in the AVOT setup uses a 5 Alternative-Forced-Choice (AFC) method to measure flicker threshold at five distinct locations namely one in the centre and each of the four quadrants parafoveally (at an eccentricity of 5° from the point of fixation) at a viewing distance of 1 m. The four quadrants were indicated as superotemporal (ST), superonasal (SN), inferonasal (IN) and inferotemporal (IT) based on the eye on which the test was carried out. The following spatiotemporal parameters were used: Temporal frequency: 15 Hz, a sensitive frequency to detect retinal deficits^9^; the size of the centre and the parafoveal stimulus was 30 and 60 min of arc respectively^10^. The starting contrast for the flicker stimulus was set at 10% and it was kept constant for all the participants. The luminance level used was 24 cd/m^2^. The target and background chromaticity were the same. The x, y chromaticity coordinates in 1931 CIE space were chosen 0.58, 0.36 to achieve a scotopic/photopic ratio of 0.7. A “demo/learning” mode should be completed (100% correct responses) by all the participants to be eligible to participate in the main test.

Participants were instructed to maintain fixation at a blinking square in the centre throughout the test. There were diagonal flankers on each of the four quadrants to keep the participants’ attention in the central region. Participants were instructed to record the location of stimulus by pressing a numeric keypad, whose elevated buttons mirrored the location of the stimuli on the screen. Participants pressed the sixth button at the top when they were not aware of the stimulus location, in which case, the software would then reassign the response to one of the five test locations. The threshold at each of the five locations is determined by five interleaved 2-down 1-up staircases with a maximum of 10 reversals, and an average of the last six reversals was considered as flicker modulation threshold (FMT)^8^.

Red/green and Yellow/blue chromatic thresholds were assessed using the Colour Assessment Diagnosis (CAD) test^11–13^, which is a part of the AVOT setup. The coloured stimuli were presented on the display monitor and the program was controlled using a laptop. The test used dynamic luminance contrast noise (± 45%) to isolate the colour signals^13^. The testing distance for CAD test is 1.4 m. The size of the target stimulus was 48 min of arc, the stimulus duration was 720 ms, and the stimulus target moved diagonally on the dynamic noise background at the rate of 4°/s. The CAD test uses a 4 Alternative-Forced-Choice (AFC) test to determine thresholds along the 16 colour directions (12 RG; 4 B-Y directions) using multiple interleaved 2-down 1-up adaptive staircases. The participant was instructed to maintain fixation on a black dot at the centre of the screen throughout the test. The participant was asked to identify the location to which coloured stimulus had moved to, using a numeric keypad that corresponded to the 4 test locations. The average of the last six reversals was taken as a threshold value, and results are displayed in CAD units^13^. 1 Standard CAD unit refers to the mean of the normal trichromat based on the normative database^12,13^. Higher CAD units indicate that a person requires higher saturation to discriminate colours or in other words, has poorer chromatic discrimination.

### Statistical analysis

All the statisical analyses in this study were performed using SPSS software (IBM SPSS, version 25; IBM Corp., Armonk, NY, USA). Shapiro–Wilk test was performed to assess for normality. The test revealed some of the visual function parameters were not normally distributed (*p* < 0.05). Therefore, to keep it consistent across all the parameters, non-parametric test was the choice of testing to assess statistical significance with a p-value of 0.05. Linear regression analysis was performed to assess how visual functions relate to visual acuity and how all the visual functions were related to foveal thickness.

## Results

Twenty eyes from 20 patients with resolved endophthalmitis were included in this study (see Table 1 for more details). All had unilateral endophthalmitis. The proportion of males was 60% (12/20). The proportion of right eyes that were tested was 55% (11/20). The participant’s age ranged from 18 to 71 years (Median age: 54 years). The median duration between clinically resolved state of endophthalmitis and date of testing was 22 months. The most frequent type of endophthalmitis was exogenous endophthalmitis (70%, 14/20), followed by endogenous endophthalmitis (30%, 6/20). The most common causative organism was bacterial 40% (7/20), followed by the fungal infection, which was 15% (3/20).

**Table 1:** Visual acuity and patient profile among the patients with resolved endophthalmitis.

Patient ID	Age (y)/gender	BCVA (logMAR)	Type of microorganism	Cause
EN001	33/M	0.08	No growth	Endogenous
EN002	33/M	0.00	Bacterial	Exogenous
EN003	44/M	0.20	No growth	Endogenous
EN004	62/M	0.30	No growth	Exogenous
EN005	58/F	0.50	No growth	Exogenous
EN006	55/F	0.24	Bacterial	Exogenous
EN007	61/M	0.60	Fungal	Exogenous
EN008	18/F	0.20	Fungal	Endogenous
EN009	65/F	0.40	Bacterial	Exogenous
EN010	52/F	0.00	No growth	Endogenous
EN011	60/F	0.50	No growth	Exogenous
EN012	53/M	0.10	Fungal	Exogenous
EN013	64/M	0.20	No growth	Exogenous
EN014	44/M	0.70	Bacterial	Exogenous
EN015	44/M	0.40	No growth	Endogenous
EN016	27/M	0.60	Bacterial	Exogenous
EN017	49/F	0.50	No growth	Exogenous
EN018	60/M	0.20	No growth	Exogenous
EN019	71/M	0.60	Bacterial	Exogenous
EN020	64/F	0.20	Bacterial	Exogenous

**Figure 2:**
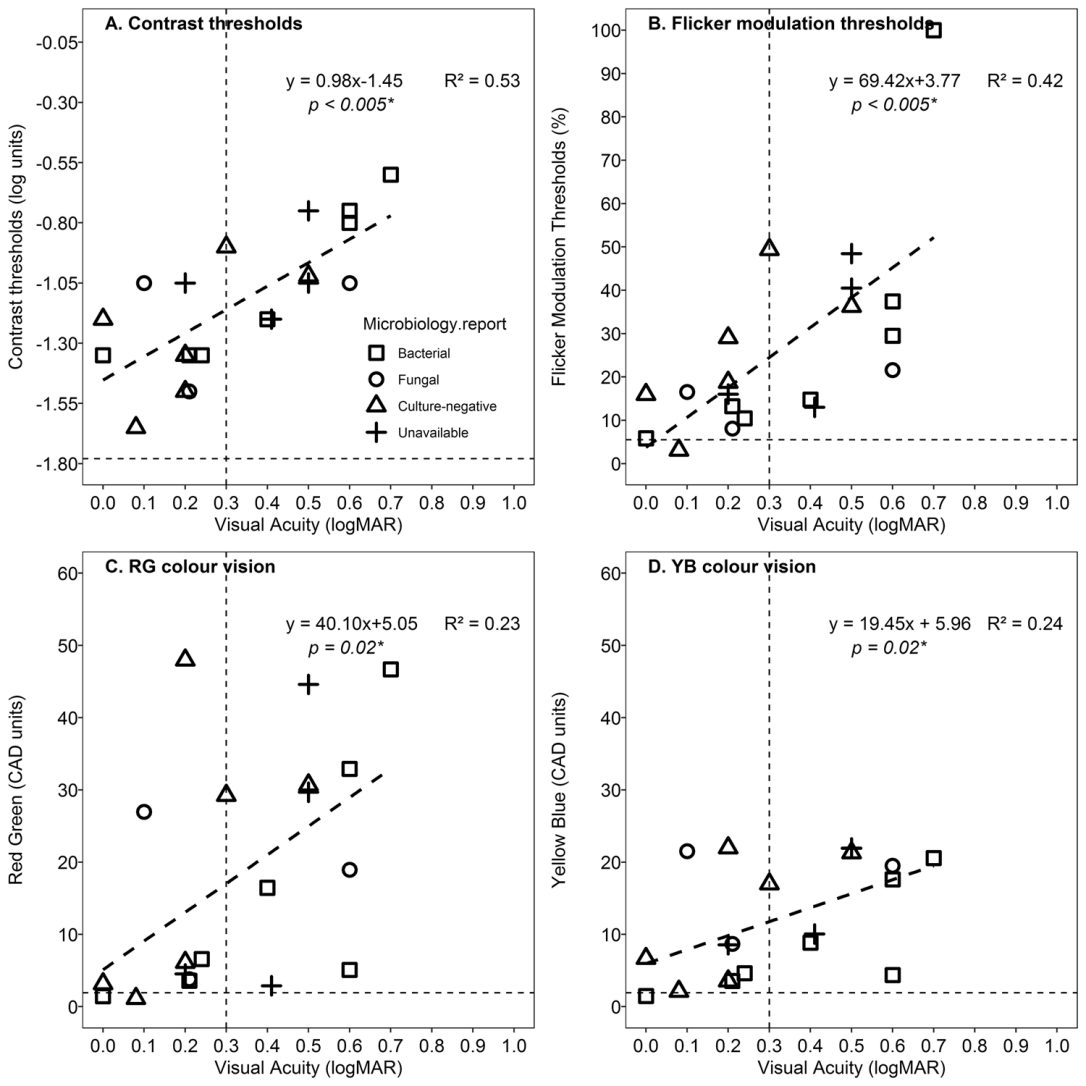
Scatter plots showing how each of the visual functions relates to visual acuity with the mapped microbiologic profile. (**A**,**B**) show spatial and temporal contrast thresholds against visual acuity. (**C**,**D**) show R-G and YB chromatic thresholds respectively plotted against VA. The black dashed lines show the regression line fitted by a linear function whose equation is provided in each of the panels. The horizontal grey dashed line in each of the panels indicates age-corrected normal values for the visual functions. The regression equation is provided in each of the panels along with coefficient of determination (R^2^). The *p* value indicates of the slope is significantly different from zero. The vertical line indicates visual acuity cut-off of 20/40 (logMAR 0.30). The legend indicates the microbiological profile of these patients as well. Three data points have been horizontally shifted by 0.01 log unit to avoid overlapping of data.

The median values for the visual functions are provided in Table 2 along with a reference for normative age-matched values for each of the visual functions^10,13–15^. The median central retinal thickness in the cohort of patients was 260 μm (IQR: 91.75 μm). The scatter plots in Fig. 2, which showed how each of the visual functions relates to visual acuity and the microbiological profile is mapped on it. Panel 2A and Panel 2B show spatial and temporal contrast thresholds against visual acuity. Panel 2C and Panel 2D show R-G and YB chromatic thresholds respectively plotted against VA. The black dashed lines show the regression line fitted by a linear function whose equation is provided in each of the panels. The horizontal grey dashed line in each of the panels indicates age-corrected normal values for the visual functions. The regression equation is provided in each of the panels along with coefficient of determination (R^2^). The p-value indicates of the slope is significantly different from zero. The vertical line indicates visual acuity cut-off of 20/40 (logMAR 0.30). The legend indicates the microbiological profile of these patients as well. Three data points have been horizontally shifted by 0.01 log unit to avoid overlapping of data.

**Table 2:** Age profile and median of the outcome variables of the patients.

Outcome variables	Patient cohort	Coefficient of variation (%)	Types of endophthalmitis		p-value	Age-matched reference values
			Endogenous (n = 5)	Exogenous (n = 15)		
Age (years)	54	N/A	38.20	55.07	0.06	N/A
Visual acuity (logMAR)	0.27	66	0.18	0.38	0.27	≤ 0.01^24^
Log contrast threshold	− 1.13	25	− 1.41	− 1.04	0.03*	− 1.78 ± 0.12^25^
Central cone FMT (%)	17.64	84	13.85	30.59	0.07	5.48 (IQR, 3.47)^^^
Red – Green (CAD units)	11.52	92	3.43	23.03	0.02*	1.45–1.91^#^
YB threshold (CAD units)	9.45	64	6.22	14.32	0.11	1.37–2.15^#^

^Refers to median value obtained from 77 normal healthy adults (Age range 35–65 years)^23^.

^#^Refers to the lower and upper limits for R-G and B-Y chromatic thresholds (Age range 35–65 years)^21^.

*p* values obtained from comparison between exogenous and endogenous endophthalmitis using independent samples Mann–Whitney test.

*Refers to the functions that are statistically significant (p < 0.05).

**Figure 3:**
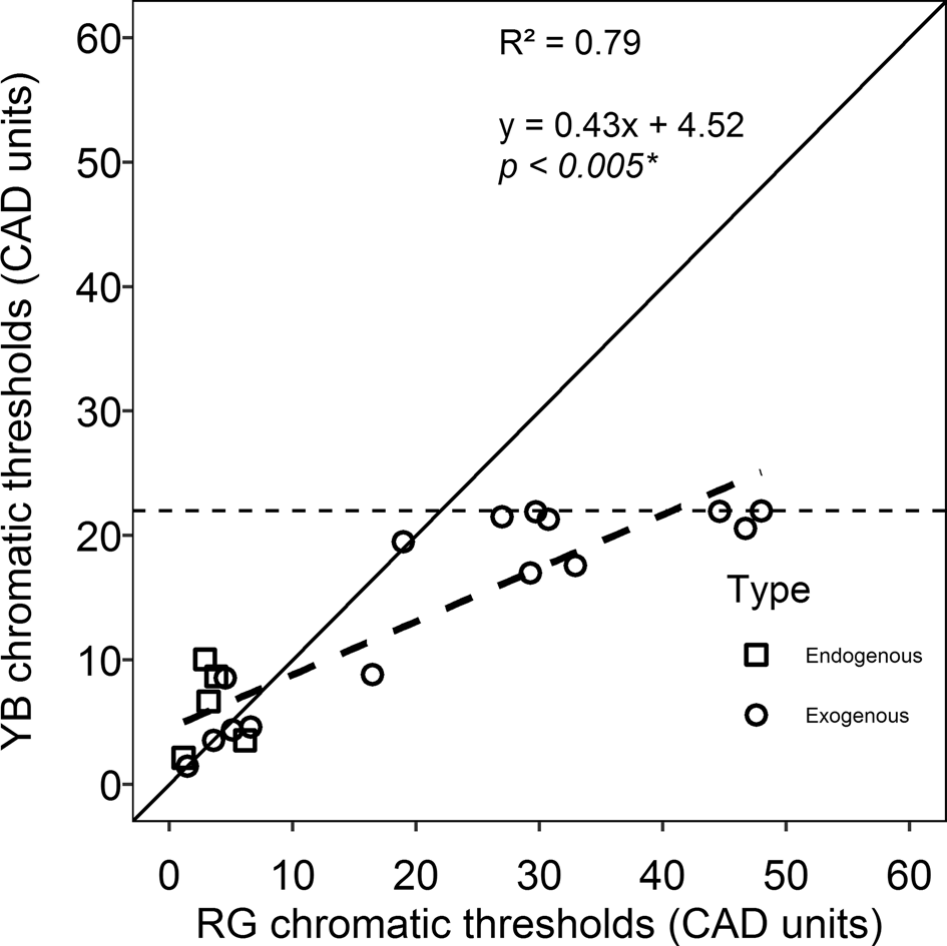
Scatterplots of YB thresholds against RG thresholds. The solid line indicates the line of equality. The dotted line show the regression line fitted by a linear function whose equation is provided. The horizontal dashed line indicates the maximum YB threshold that can be measured using the instrument.

The visual acuity was better than 0.30 (20/40) in all the patients except one in the culture-negative group (Fig. 2). Three out of 7 patients with bacterial infection had a visual acuity of 20/80 or worse. Non-parametric group-wise analysis was done to compare the FMTs across four test locations (superotemporal, superonasal, inferotemporal and inferonasal), and it revealed there were no significant differences between the four quadrants (p = 0.78). A Mann–Whitney independent test revealed that there was no statistically significant difference between RG and YB CAD thresholds (p = 0.61). However, the percentage of subjects in which RG thresholds were higher than YB was 65% (13/20) as shown in Fig. 3. The solid line indicates the line of equality. The dotted line show the regression line fitted by a linear function whose equation is provided. The horizontal dashed line indicates the maximum YB threshold that can be measured using the instrument.

**Figure 4:**
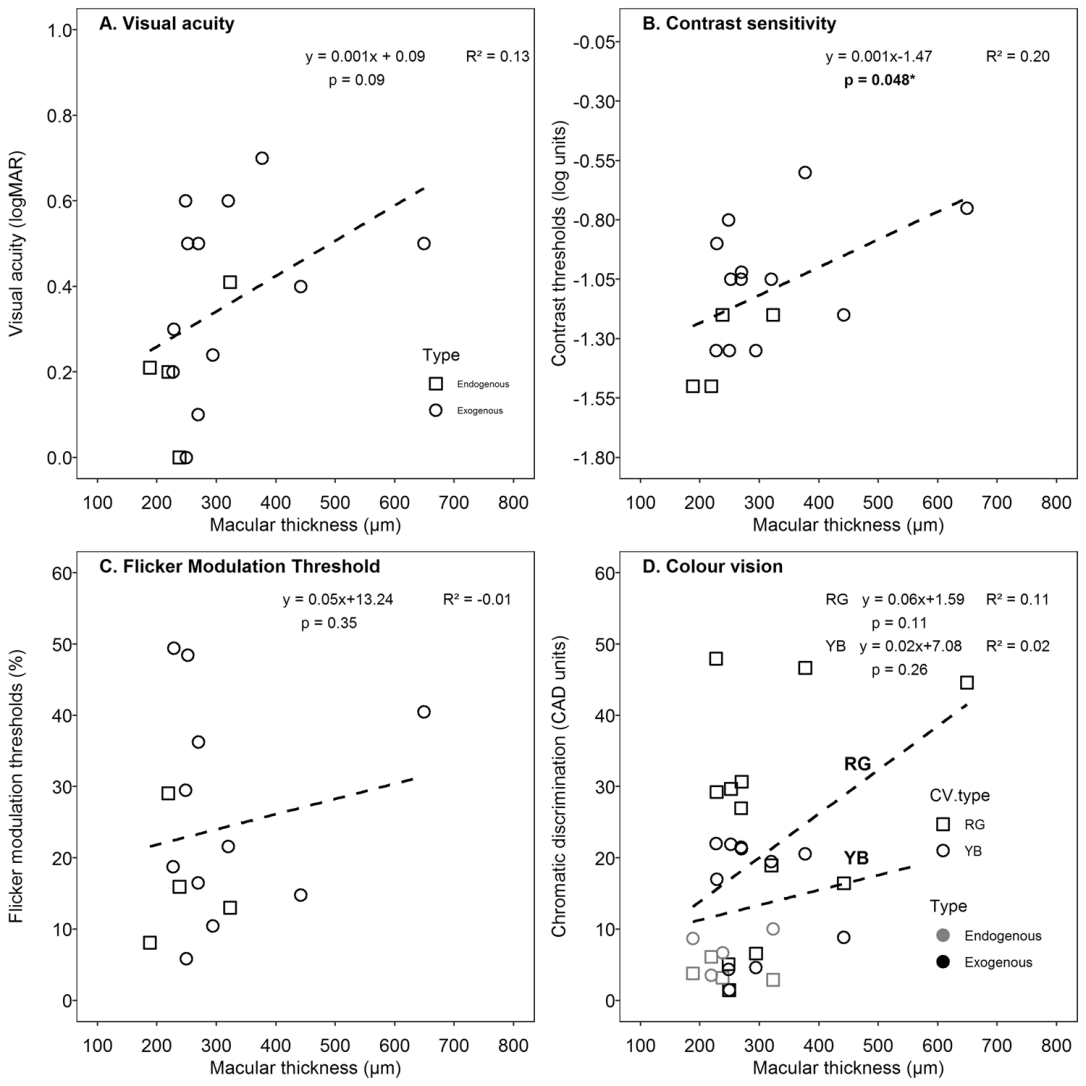
Scatterplots of visual functions against the foveal thickness. (**A**,**B**) shows visual acuity, and log contrast thresholds plotted against central macular thickness. (**C**) Show flicker modulation thresholds plotted as a function of central macular thickness. Different shape symbol in the panels (**A**–**C**) indicate the exogenous (open circles) and endogenous (open squares) types of endophthalmitis. (**D**) Show R-G and B-Y chromatic thresholds plotted against macular thickness. The different shape symbols in the panel indicate RG and RB colour vision. The black and grey symbols refers to exogenous and endogenous endophthalmitis respectively. The dashed lines show the regression line fitted by a linear function whose equation is provided in each of the panels.

However, only differences noted *in contrast thresholds and RG thresholds* were statistically significant (p = 0.03 and p = 0.02 respectively). None of the visual functions shows significant correlation with respect to age (p > 0.05), except RG CAD thresholds (Spearman correlation, ρ = 0.47, p = 0.036). Visual functions were plotted as a function of foveal thickness (Fig. 4). Panel 4A and 4B shows visual acuity, and log contrast thresholds plotted against central foveal thickness. Panels 4C show flicker modulation thresholds plotted as a function of central foveal thickness. Different shape symbols in the panels 4A-C indicate the exogenous and endogenous types of endophthalmitis. Panel 4D shows R-G and B-Y chromatic thresholds plotted against foveal thickness. The different shape symbols in the panel indicate exogenous and endogenous types of endophthalmitis. The dotted lines show the regression line fitted by a linear function whose equation is provided in each of the panels.

**Figure 5:**
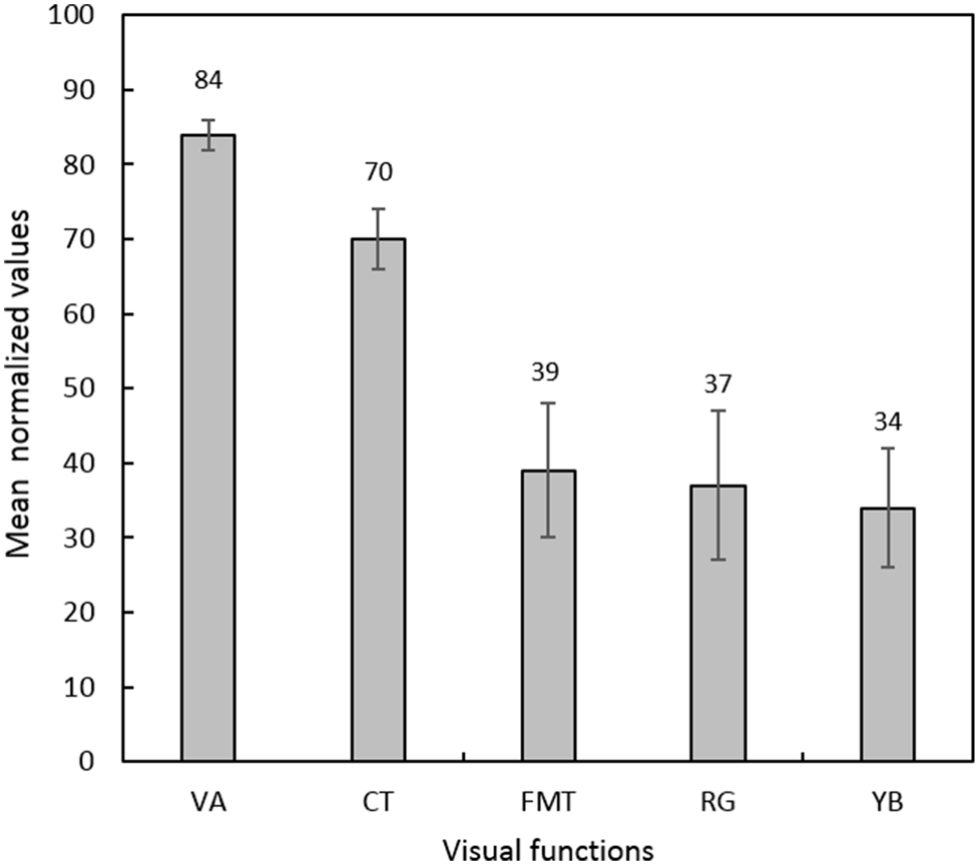
Bar diagram showing mean normalised visual functions (with SEM) in comparison to respective age-matched normative values. Visual acuity (VA), contrast threshold (CT), flicker modulation threshold (FMT), red/green (RG) and yellow/blue (YB) sensitivities are normalised with their respective age-matched values. 100% refers to equal to age-matched normative values. The error bars represent the standard error of the mean. The order of deficits in the visual functions in ascending order was VA < CT < FMT < RG < YB.

Only contrast sensitivity (Panel Fig. 4B) showed a significant relationship as revealed by linear regression (p = 0.048). Flicker and chromatic thresholds showed a poor relationship with foveal thi*ckness*. *Visual acuity and contrast thresholds*, worsened significantly with an increase in macular thickness (Spearman correlation, ρ = 0.56, p = 0.03 and ρ = 0.52, p = 0.04 respectively). The summary of visual functions post-recovery is captured in Fig. 5. Visual acuity (VA), contrast threshold (CT), flicker modulation threshold (FMT), red/green (RG) and yellow/blue (YB) sensitivities are normalised with their respective age-matched values. 100% refers to equal to age-matched normative values. The error bars represent the standard error of the mean. The order of deficits in the visual functions in ascending order was VA < CT < FMT < RG < YB.

Visual acuity remained the least affected visual function, followed by contrast sensitivity, flicker modulation threshold, red/green and yellow blue colour vision. The time for completion of FMT was about 15 min, whereas time taken for CAD test was ~ 25–30 min. Visual acuity and contrast sensitivity typically took about ~ 3 to 5 min each for completion.

## Discussion

To the best of our knowledge, this is the first study to report deficits in contrast, colour discrimination and flicker thresholds in patients who have recovered from endophthalmitis. The study reports three key findings. Firstly, visual functions including contrast sensitivity, flicker and chromatic sensitivity were poorer despite relatively good VA. Secondly, red-green colour vision losses greater than yellow-blue are more frequently observed.

Fifty-five per cent (11/20) of the patients had a visual acuity ≤ 0.3 logMAR, which is considered as a good visual outcome in endophthalmitis^1^. Among, those who had visual acuity of ≤ 0.3, none of the patients had a log contrast sensitivity higher than 1.66 (lower limit for age-matched normal values)^15^ Only one patient (10%) had a lower threshold than age—matched cone flicker threshold values^10^. Only 18% (2/11) of the patients had CAD thresholds (RG and B-Y) and FMTs lower than the upper limits for the age-matched database^10–13^. These results indicate that the visual acuity is fairly preserved, however, the visual functions such as contrast sensitivity, chromatic discrimination and cone-mediated FMT remain impaired. These findings bear resemblance with the study that reported that RG and YB colour vision and pupillary functions remain impaired despite good VA recovery in patients with optic neuritis^16,17^. Although the cohorts may be different, it gives insight that different visual functions can recover at varying time points. Another potential explanation for this differential performance in visual functions could be as follows: Endophthalmitis might be considered as a form of retinal trauma in that damage to cells will release cytokines and likely will result in the phenomenon of spreading depolarization^18–20^. Retinal trauma is associated with a widespread disjunction of the bipolar-photoreceptor synapse extending far beyond the area of even a localized retinal detachment^21–23^. This phenomenon may underlie, in part, the responsible for global level changes (e.g., color vision) despite preserved high contrast central vision.

In addition, we also explored how visual outcomes are associated with microbiological samples obtained from the patients (Fig. 2). The culture-negative group (83%; 5/6) showed VA of at least 20/40 or better compared to culture-positive groups (50%; 5/10). This is consistent with the endophthalmitis vitrectomy study that found that there is 80% of patients had VA 20/100 or better in culture-negative groups compared to about 50% in patients with the culture-positive groups^24^.

There are no previous studies that have reported spatial/temporal contrast sensitivity or colour vision in the cohort of cases with resolved endophthalmitis. Therefore, we cannot directly compare these results with any other previous study. However, it is important to note that all visual functions exhibited wide inter-observer variation, except contrast thresholds (indicated by coeffficient of variation in Table 2). The low inter-observer variability may indicate the robustness of the contrast measure as well as the ability to detect functional deficits compared to age-matched norms. This inference is supported by a few studies that have used spatial contrast thresholds as a useful tool to quantify the functional vision changes in patients with posterior vitreous detachment (PVD) and with vitreous floaters^25–28^. Contrast threshold measures significantly improved post-vitrectomy following PVD^25^. Although the patient cohorts are different, the usefulness of contrast thresholds to capture retinal functions indicate that it might be a sensitive marker to measure residual functional vision in patients with resolved endophthalmitis.

Although, the finding that the magnitude of RG loss was not significantly different from YB loss, the number of individuals who had larger RG loss compared to YB loss seems to agree with the Kollner’s rule^29^, which states that in a generalized retinal disease, the expected magnitude of B-Y loss is more significant than RG loss. This inference based on the assumption the retinal findings often found in the patients in resolving endophthalmitis can be classified as a generalized retinal disease. However, the mechanism of the colour vision loss remains unclear. Nevertheless, it can be hypothesized that the locus for colour vision loss is likely due to the cone photoreceptors (L, M and S cones) loss, rather than post-retinal circuitry of the parvocellular and koniocellular pathway which is responsible for RG and YB colour vision respectively^30^.

Lastly, the significant relationship between contrast threshold and retinal thickness suggests there might be an association (Fig. 4B). However, both VA and CT show significant correlation with foveal thickness indicating structure–function relationship. Previously, it has been shown visual acuity was significantly worse in the presence of atrophy to inner retinal layers and inner segment ellipsoid disruption^3^. The exact mechanism why visual functions deteriorate with an increase in foveal thickness remains unclear as there is no evidence to suggest that macular edema or thickening leading to a poorer visual function than retinal thinning.

The current study has some limitations. The flicker thresholds were limited only to the central 5^o^ because, we had normative database for that eccentricity^10^ and it is therefore easy to compare it against the database to make sure that flicker thresholds were not age-related changes. The visual function characterization of subtypes of infectious endophthalmitis (bacterial/fungal) was not done extensively, owing to the small sample size in each subgroup. Besides, this is not a longitudinal study; only associations and not causations can be made between the visual functions and structural changes in the retina. Future longitudinal studies with multiple follow-ups will be useful to track the structure–function relationship more robustly. The time taken for testing can be considerably reduced by modification of the starting contrast values for the flicker and CAD tests. The fact that these tests are able to identify the deficits allows us to do further customization that are needed to make the tests quicker without sacrificing sensitivity and specificity.

Although, these parameters may not be an immediate marker used in the management of endophthalmitis in the acute phase. However, these are vital markers of the common visual functions that reveal the overall quality of vision of the patient who has recovered from endophthalmitis. This is an important information for a treating ophthalmologist to manage the patients more efficiently.

## Conclusions

To conclude, we recommend to include measurement of contrast sensitivity in the routine clinical examination, in addition to the visual acuity in patients who have resolved endophthalmitis because contrast thresholds exhibit low interobserver variability and a strong association with central retinal thickness. Thus, contrast sensitivity may capture the functional deficits experienced by the patients daily.
